# Incidence trends and survival analysis of enteropathy-associated T-cell lymphoma

**DOI:** 10.3389/fimmu.2025.1605542

**Published:** 2025-09-25

**Authors:** Lina Xiang, Tianen Pan, Xiaoying Man, Qiang Zhuang

**Affiliations:** ^1^ Department of Emergency, The First Affiliated Hospital of Wenzhou Medical University, Wenzhou, China; ^2^ Department of Hematology, The First Affiliated Hospital of Wenzhou Medical University, Wenzhou, China

**Keywords:** enteropathy-associated T-cell lymphoma, conditional survival, nomogram, prognosis, SEER

## Abstract

**Background:**

Enteropathy-associated T-cell lymphoma (EATL) is a rare, aggressive form of T-cell non-Hodgkin lymphoma, primarily affecting the small intestine. Due to its extremely low incidence and poor prognosis, studies on EATL are limited. This study aims to analyze the incidence trends, prognostic factors, and survival outcomes of EATL using the SEER database.

**Methods:**

We analyzed the incidence trends of EATL using SEER data from 2000 to 2021 and performed multivariate Cox regression to identify independent prognostic factors. Conditional survival (CS) analysis was performed to evaluate survival outcomes, and a CS-nomogram was developed to predict 1-, 3-, and 5-year overall survival (OS) and 5-year CS.

**Results:**

The age-adjusted incidence rate of EATL was 0.014 per 100,000, showing a significant upward trend (APC 2.63). Multivariate analysis identified age, tumor site, tumor stage, surgery, and chemotherapy as independent OS risk factors. CS analysis showed that the 5-year survival probability progressively increased from 16% to 48%, 67%, 83%, and 93% after surviving 0, 1, 2, 3, and 4 years post-diagnosis. The dynamic nomogram showed excellent performance in predicting survival and stratifying patients into risk groups.

**Conclusions:**

This study provided valuable insights into the epidemiology, prognosis, and survival outcomes of EATL. The increasing incidence and CS outcomes highlighted the importance of early detection and intervention. The dynamic nomogram model developed here offered a more accurate prediction of individual survival outcomes and can aid in clinical decision-making.

## 1 Introduction

Enteropathy-associated T-cell lymphoma (EATL) is a rare and aggressive T-Cell Non-Hodgkin lymphoma that primarily arises in the small intestine and is closely associated with celiac disease (1–4). The incidence of EATL remains low, making it a challenging entity to study. The prognosis of EATL is generally poor, with reported median 5-year overall survival (OS) ranging from 0% to 59% (5). In patients with EATL who have a prior diagnosis of refractory celiac disease, the 5-year OS is significantly lower, ranging from 0% to 8% (5–9). Treatment for this disease typically involves surgery and chemotherapy, but the prognosis remains discouraging, with OS rates being dismal, particularly in advanced stages (1, 10, 11). Given the rarity of this malignancy, large-scale studies to fully understand its epidemiology, prognostic factors, and long-term survival outcomes remain sparse. Therefore, comprehensive studies of EATL are crucial to improve clinical management and patient outcomes.

Despite the recognition of EATL’s aggressive nature, studies investigating its survival prognosis have been limited, and available data are mostly derived from case reports or small series. Also, current survival models often fail to address the dynamic nature of survival outcomes, particularly in terms of changing prognostic factors over time. In this study, we compare the advantages of conditional survival (CS) analysis to traditional survival analysis, which allows for a more nuanced view of survival. CS analysis can identify critical time points post-diagnosis where mortality risk is highest, thus offering valuable insights into the timing of intervention and patient management (12–15).

To address these gaps in research, we leveraged the SEER database, which provides a large cohort with long-term follow-up data. The SEER database is invaluable for studying rare cancers like EATL, as it offers a robust sample size that allows for more reliable analysis of epidemiological trends, survival outcomes, and the development of novel prognostic models. In this study, we aim to evaluate the incidence trends and use CS analysis to better understand the epidemiological information and survival prognosis of EATL patients. Additionally, we developed a novel nomogram model based on these dynamic survival factors to provide more accurate outcome predictions.

## 2 Methods

### 2.1 Patient cohort

We analyzed data from the SEER database, which annually collects information on patient demographics, tumor morphology, stage, site, first course of treatment, and vital status from population-based cancer registries across the United States. Covering approximately 48% of the U.S. population, the SEER database is publicly accessible. The data used in this study were obtained from SEER∗Stat (version 8.4.4). Inclusion criteria for patients were as follows: (1) diagnosis year between 2000 and 2021, and (2) ICD-O histology/behavior codes: 9717/3, which corresponds to “Enteropathy-associated T-cell lymphoma.” For the prognostic analysis, our study excluded patients with unknown follow-up data or unclear surgical treatment information.

### 2.2 Variables of interest and primary outcome

We collected information on patient demographics, tumor characteristics and treatment information, including age at diagnosis, sex, and race, tumor site, tumor stage at diagnosis, co-existing with multiple tumors, surgery, radiotherapy, chemotherapy, vital status (dead or alive and cause of death) and survival time. The primary outcome analyzed in this study was OS, which was defined as the time from diagnosis to death from any cause or the last follow-up.

### 2.3 Statistics

#### 2.3.1 Incidence

To assess incidence, we computed the age-adjusted incidence rate (per 100,000 person-years) using the SEER*Stat software, based on the 2000 US Standard Population. The annual percent change (APC) was determined through joinpoint regression models, utilizing Joinpoint software (16).

#### 2.3.2 Prognostic factor analysis

Prognostic factor analysis was conducted by selecting relevant clinical variables and evaluating their association with survival outcomes using multivariate Cox regression analysis. Significant factors are identified based on P-values and hazard ratios.

#### 2.3.3 Conditional survival analysis

We introduced a CS analysis based on OS, which was defined as the probability of survival at a given time point, taking into account the time elapsed since diagnosis and the patient’s survival up to that point. The CS probability at time t+t_0_ was calculated as the probability that a patient who had already survived up to time t_0_ (e.g., 1 year, 3 years, etc.) and continue to survive beyond t_0_. It’s computed as: CS(t∣t_0_)=S(t+t_0_)/S(t_0_), where S(t+t_0_) is the overall survival function at time t+t_0_ and S(t_0_) is the overall survival function at the initial time point t_0_ (13).

#### 2.3.4 Nomogram model construction

We randomly divided the entire cohort into a training group and a validation group at a 7:3 ratio for model development and validation. Several studies have demonstrated the superiority of the random survival forests (RSF) algorithm over traditional Cox regression for variable selection (17, 18). The RSF method excels at handling complex relationships between variables, accounting for non-linear effects, and eliminating the need for pre-selection of variables. Therefore, in our study, patient data in training cohort, with relevant clinical features identified through the RSF algorithm as potential prognostic factors. Variables with a variable importance (VIMP) score greater than 0.01 were considered significant prognostic factors and included in the final model. The Cox proportional hazards model was employed to integrate CS probabilities at various time points, facilitating the development of the CS-integrated nomogram model for dynamic survival predictions. The model’s predictive performance was evaluated using several metrics in both training and validation cohorts, including the calibration curves, and ROC curve analysis, to assess its accuracy and discriminatory ability. Additionally, the clinical utility of the model was evaluated through decision curve analysis (DCA), which assessed the net benefit of the model across different threshold probabilities, helping to determine its practical value in clinical decision-making.

Finally, we calculated the total score for each patient based on the risk values assigned to the variables in the nomogram. The cumulative score was then used to classify patients into different risk categories (e.g., low and high risk). This stratification aided in predicting individual survival probabilities, guiding treatment decisions, and optimizing patient management according to their predicted risk.

All statistical analyses were performed using R software. A P-value of <0.05 was considered statistically significant.

## 3 Results

### 3.1 Patient characteristics

In our study investigating predictive factors, we analyzed baseline demographic and clinical characteristics to evaluate their significance. **Table 1** presents the demographic and clinical features of the overall cohort (n=401). The cohort included patients of various ages, with the largest group being aged 60–69 years (30.7%). The majority of patients were male (57.9%) and white (72.8%). Tumors were most commonly located in the small intestine (75.3%), followed by the gastrointestinal tract, NOS (14.2%), and lymph nodes (10.5%). Regarding tumor stage, the majority of patients had distant metastasis (35.2%), while 33.9% had localized tumors. Most patients had solitary EATL (79.8%) and underwent surgery (68.8%). Only 2% received radiotherapy, while 56.9% received chemotherapy.

**Table 1:** Demographic and clinical characteristics of EATL patients.

Characteristics	Overall	Training	Validation
	n=401	n=280	n=121
Age at diagnosis			
<60	124 (30.9%)	86 (30.7%)	38 (31.4%)
60-69	123 (30.7%)	87 (31.1%)	36 (29.8%)
70-79	95 (23.7%)	67 (23.9%)	28 (23.1%)
≥80	59 (14.7%)	40 (14.3%)	19 (15.7%)
Sex			
Male	232 (57.9%)	164 (58.6%)	68 (56.2%)
Female	169 (42.1%)	116 (41.4%)	53 (43.8%)
Race			
White	292 (72.8%)	210 (75.0%)	82 (67.8%)
Others	109 (27.2%)	70 (25.0%)	39 (32.2%)
Tumor site			
Small intestine	302 (75.3%)	210 (75.0%)	92 (76.0%)
Gastrointestinal tract, NOS	57 (14.2%)	42 (15.0%)	15 (12.4%)
Lymph nodes	42 (10.5%)	28 (10.0%)	14 (11.6%)
Tumor stage			
Localized	136 (33.9%)	95 (33.9%)	41 (33.9%)
Regional	88 (21.9%)	61 (21.8%)	27 (22.3%)
Distant	141 (35.2%)	95 (33.9%)	46 (38.0%)
Unknown	36 (9.0%)	29 (10.4%)	7 (5.8%)
Co-existing multiple tumors			
No	320 (79.8%)	221 (78.9%)	99 (81.8%)
Yes	81 (20.2%)	59 (21.1%)	22 (18.2%)
Surgery			
No	125 (31.2%)	97 (34.6%)	28 (23.1%)
Yes	276 (68.8%)	183 (65.4%)	93 (76.9%)
RT			
No	393 (98.0%)	275 (98.2%)	118 (97.5%)
Yes	8 (2.0%)	5 (1.8%)	3 (2.5%)
CT			
No	173 (43.1%)	127 (45.4%)	46 (38.0%)
Yes	228 (56.9%)	153 (54.6%)	75 (62.0%)
Household income			
<80000$	216 (53.9%)	150 (53.6%)	66 (54.5%)
≥80000$	185 (46.1%)	130 (46.4%)	55 (45.5%)

EATL, Enteropathy-Associated T-cell Lymphoma; NOS, not other specific; RT, radiotherapy; CT, chemotherapy.

### 3.2 Incidence

**Figure 1:**
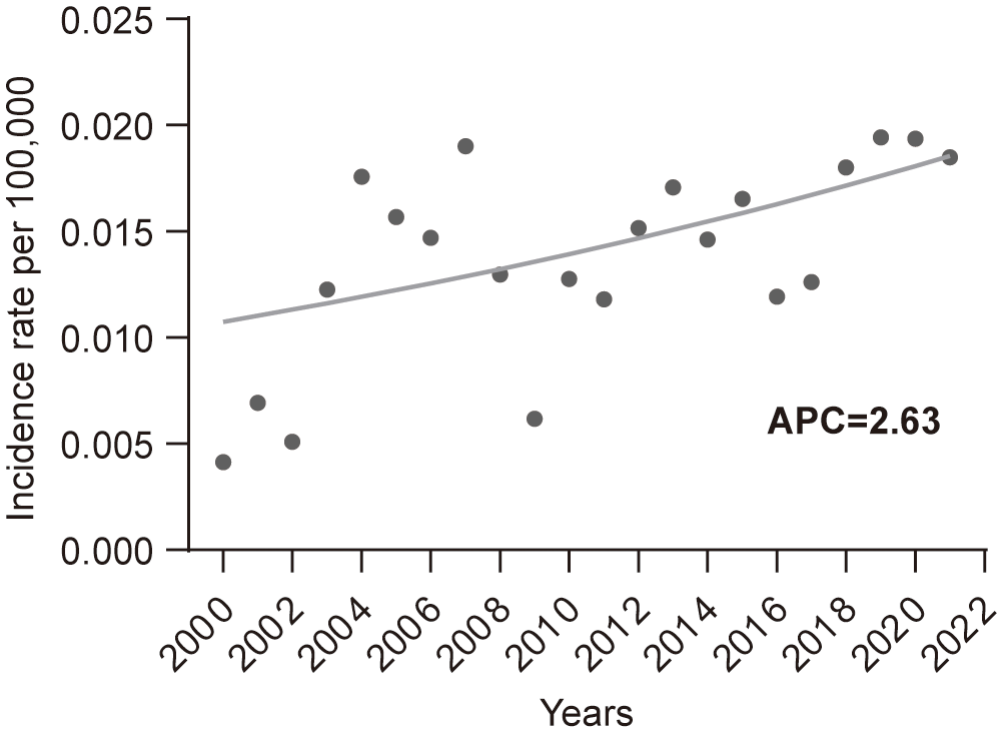
Incidence trends of enteropathy-associated T-cell lymphoma from 2000 to 2021. APC, annual percent change.

From 2000 to 2021, the age-adjusted incidence rate of EATL per 100,000 people was 0.014. During this period, the incidence of EATL showed an upward trend, with an APC of 2.63 (P <0.05, **Figure 1**).

### 3.3 Prognostic factors identification

**Figure 2:**
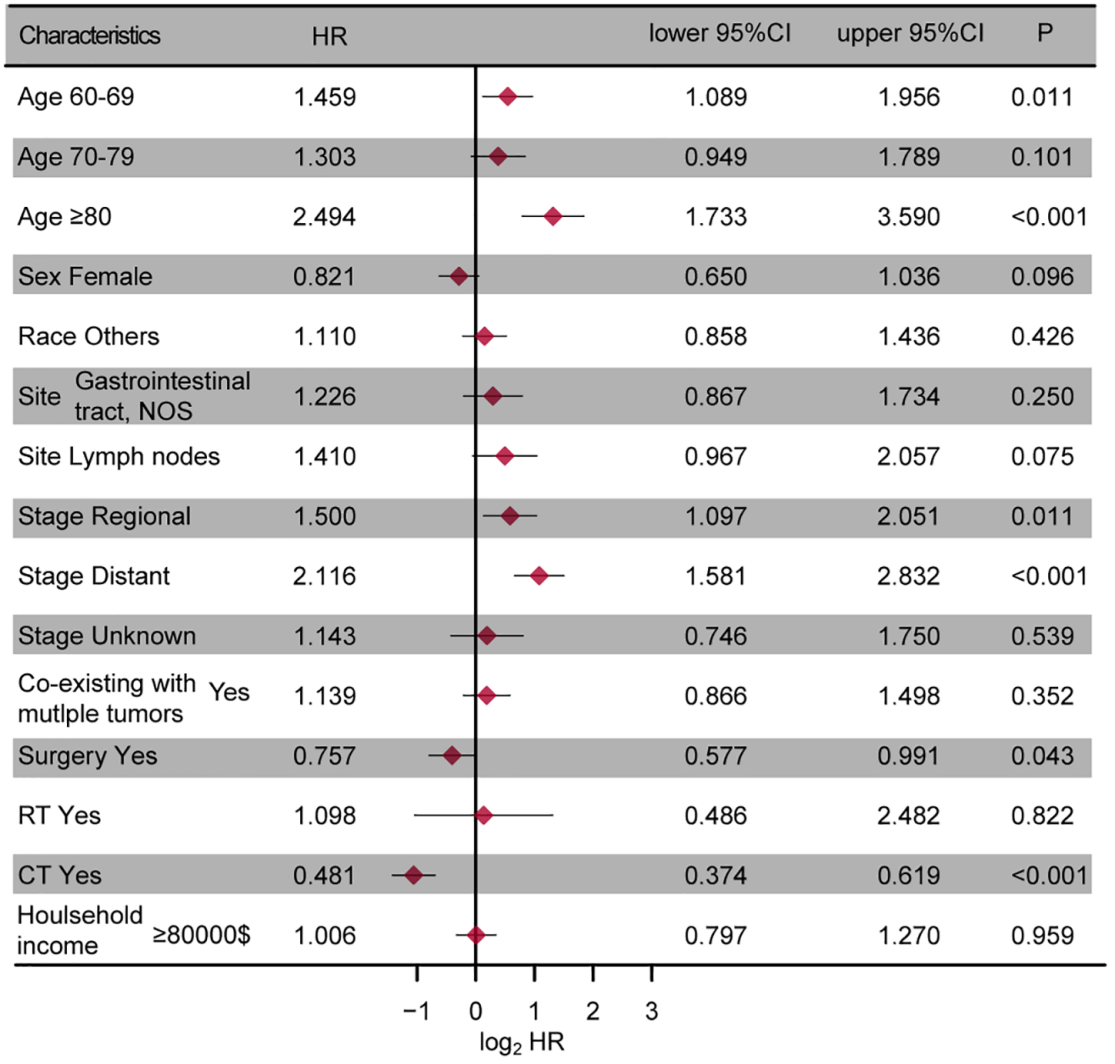
The forest plot presenting the results of the multivariate regression analysis of enteropathy-associated T-cell lymphoma. NOS, not other specific; RT, radiotherapy; CT, chemotherapy.

A multivariate Cox regression analysis was performed to identify prognostic factors in the entire cohort, with a significance level set at P < 0.05. The results were shown in **Figure 2**. The analysis revealed that age, tumor stage, surgery, and chemotherapy may serve as independent risk factors for OS (all P < 0.05), while tumor site approached borderline significance (P=0.075).

### 3.4 Survival analysis

**Figure 3:**
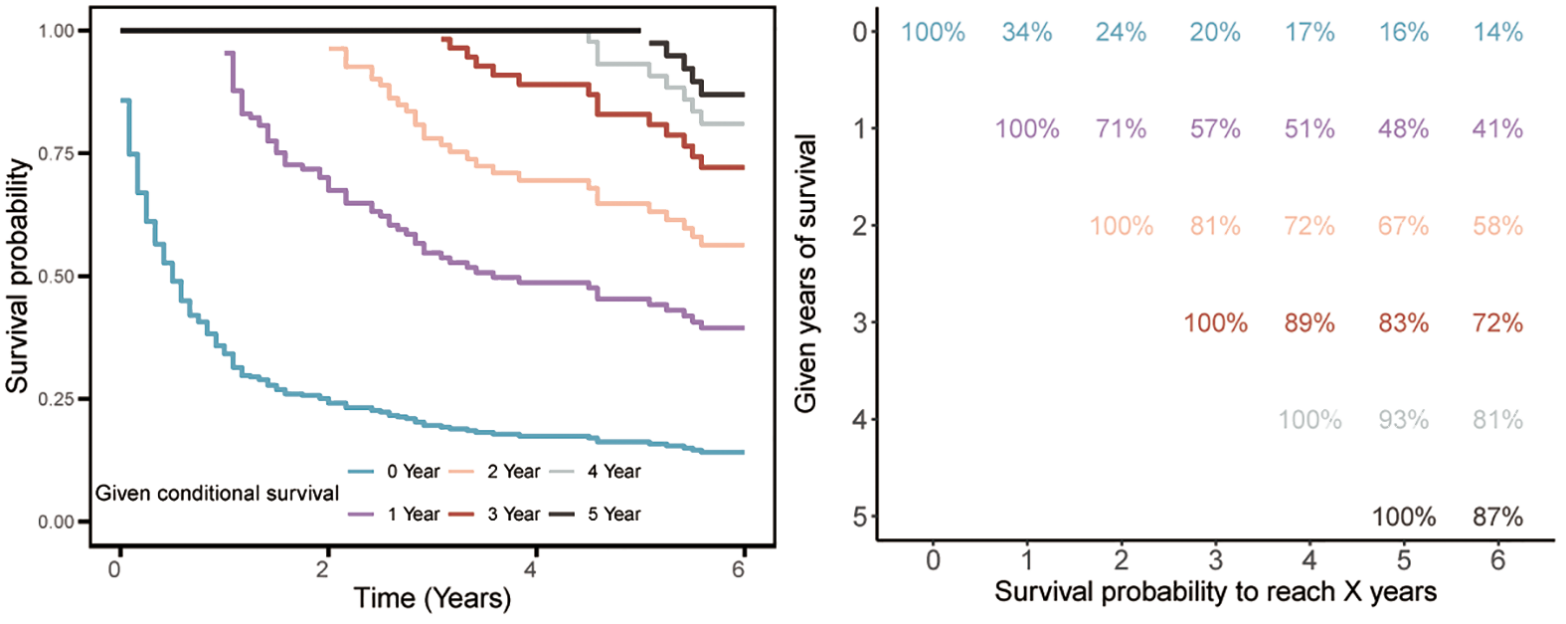
Conditional survival of enteropathy-associated T-cell lymphoma.

We further evaluated the survival prognosis of this tumor. Kaplan-Meier survival analysis revealed that the 1-, 3-, and 5-year survival rates were 34%, 20%, and 16%, respectively, indicating poor survival outcomes. Subsequent CS analysis showed that the 5-year survival probability progressively increased from 16% to 48%, 67%, 83%, and 93% after surviving 0, 1, 2, 3, and 4 years post-diagnosis, respectively (**Figure 3**). Moreover, the additional one-year survival rates after surviving 1 to 4 years were 34%, 71%, 81%, 89%, and 93%, respectively (**Figure 3**).

### 3.5 Conditional survival-based nomogram construction

**Figure 4:**
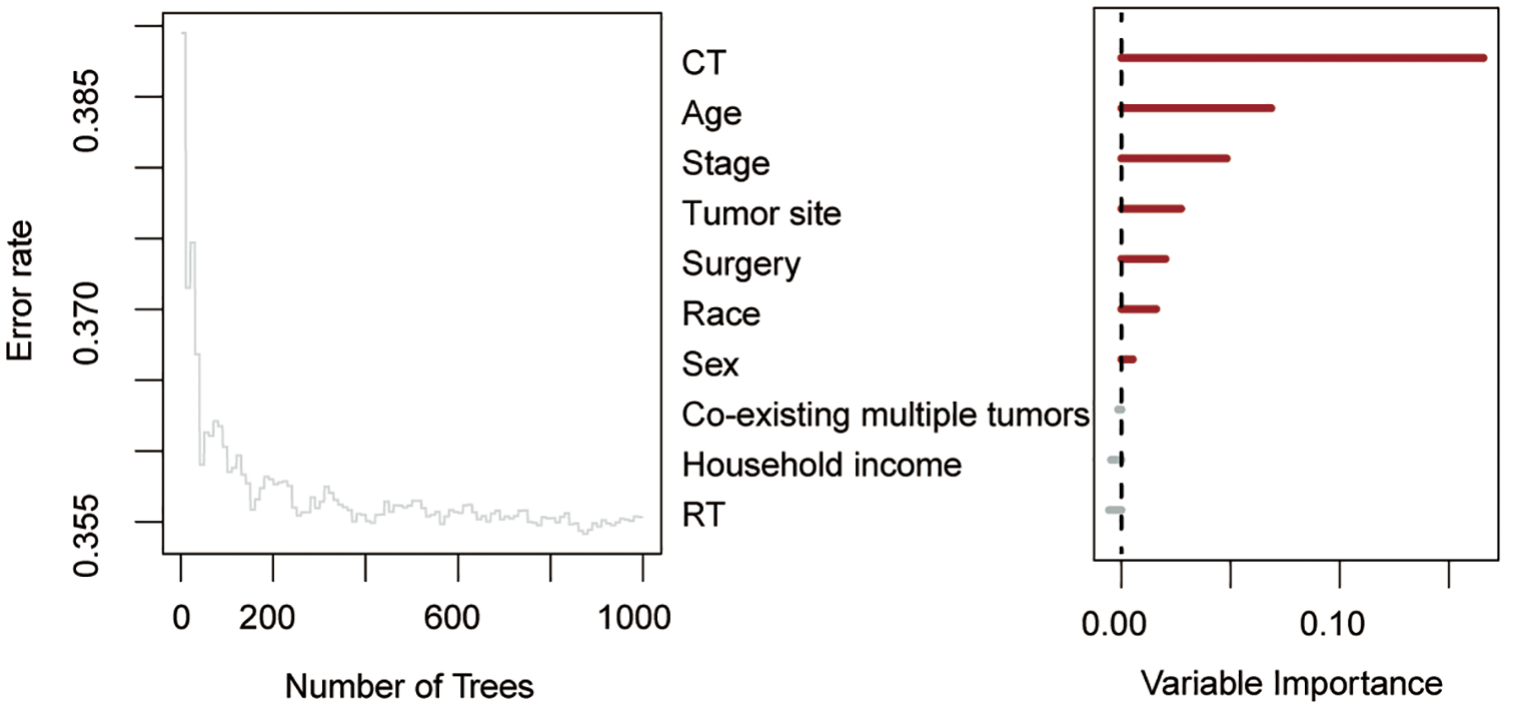
Random survival forest algorithm for selecting prognostic factor combinations. RT, radiotherapy; CT, chemotherapy.

**Figure 5:**
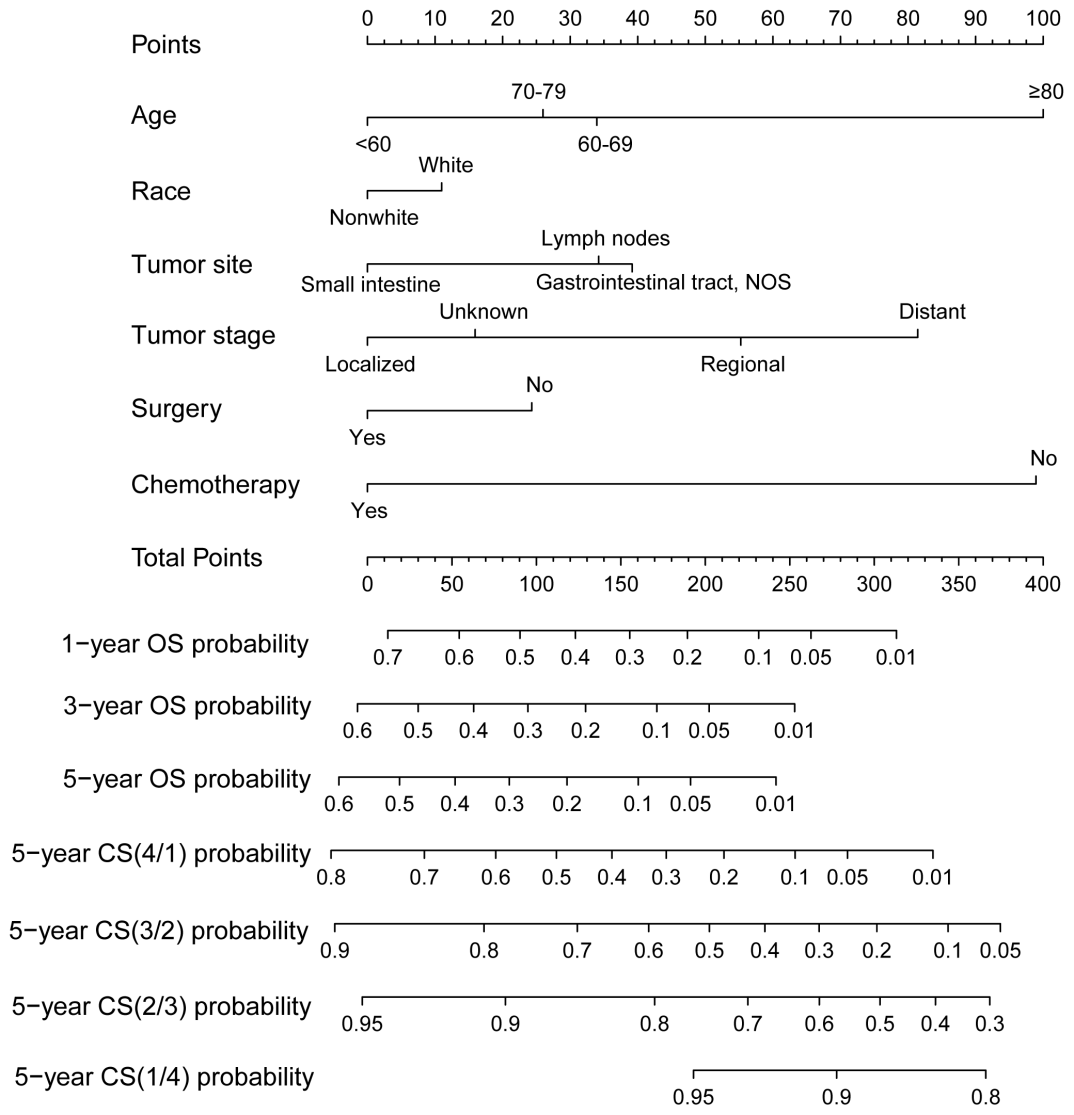
A novel conditional survival-based nomogram model for predicting 1-, 3-, and 5-year overall survival and 5-year conditional survival.

**Figure 6:**
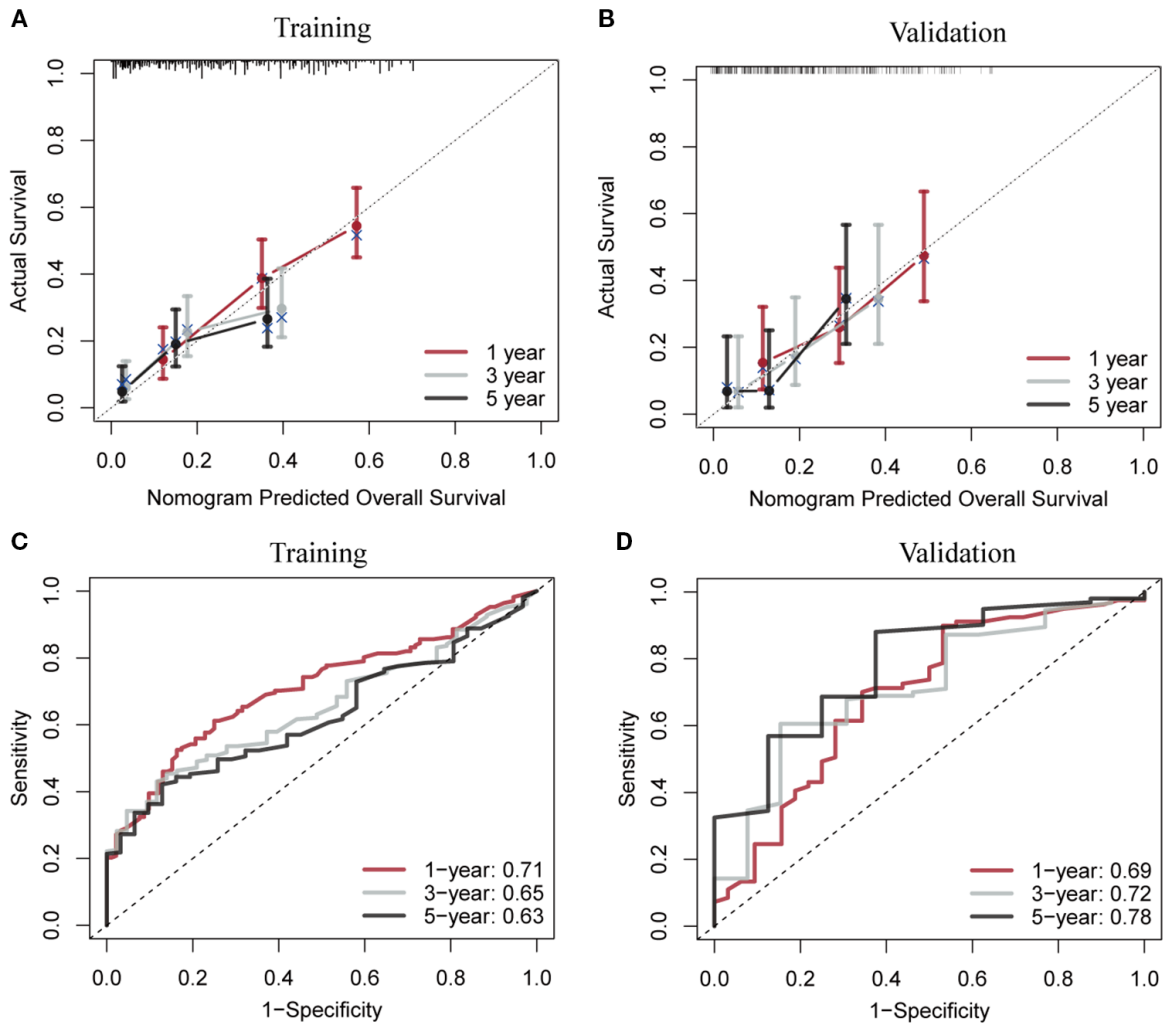
Calibration curves and receiver operating characteristic curves with area under the curve values for the conditional survival nomogram in both the training **(A, C)** and validation **(B, D)** cohorts.

**Figure 7:**
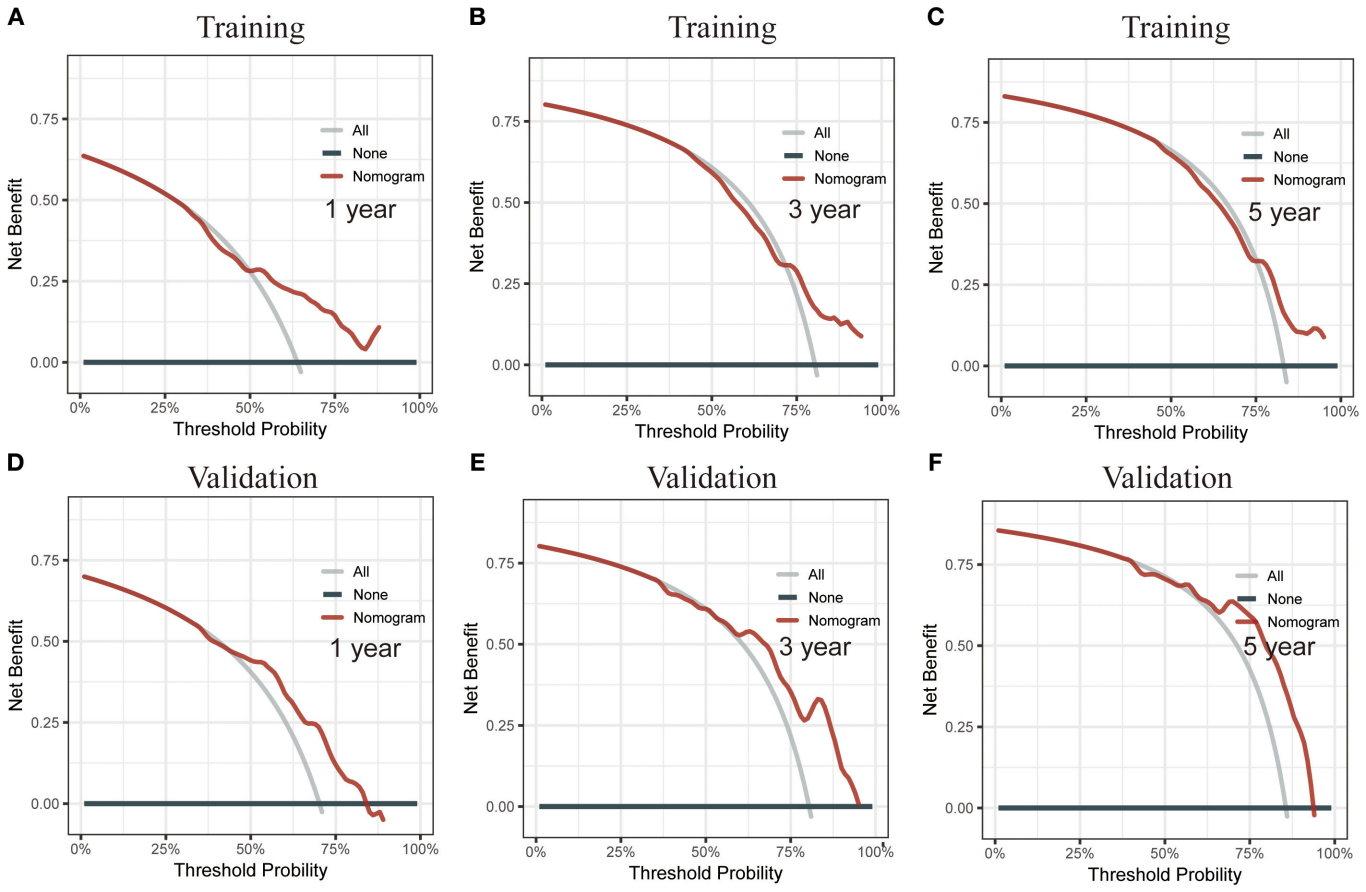
Decision curve analysis (DCA) of the CS nomogram. **(A–C)** DCA for the training cohort, showing the net benefit of the nomogram at different threshold probabilities. **(D–F)** DCA for the validation cohort, reinforcing the clinical utility of the model by illustrating its net benefit across a range of threshold probabilities.

Using RSF for variable selection in nomogram construction reduces subjectivity, effectively handles missing data, and improves model accuracy by capturing variable interactions, thereby enhancing predictive performance in survival analysis. First, the entire cohort was randomly divided into a training group and a validation group at a 7:3 ratio (**Table 1**). We then used the RSF algorithm in the training group to select prognostic variables. By setting a threshold of VIMP > 0.01, we identified age, race, tumor site, tumor stage, surgery, and chemotherapy as the final set of variables for model development (**Figure 4**). Using Cox regression and CS algorithms, we successfully developed a novel nomogram model to predict 1-, 3-, and 5-year OS and 5-year CS (**Figure 5**). We further evaluated the model’s performance in both the training and validation groups using a series of assessment metrics. The calibration curve demonstrated good agreement between the predicted and observed survival probabilities in both training (**Figure 6A**) and validation (**Figure 6B**) cohorts. The ROC curve analysis showed AUC values of 0.71, 0.65, and 0.63 for the training group (**Figure 6C**), and 0.69, 0.72, and 0.78 for the validation group (**Figure 6D**), indicating excellent discriminatory ability. Additionally, DCA further highlighted the model’s strong clinical applicability in both training (**Figures 7A–C**) and validation (**Figures 7D–F**) cohorts, demonstrating its net benefit across a range of threshold probabilities and reinforcing its value in guiding clinical decision-making.

**Figure 8:**
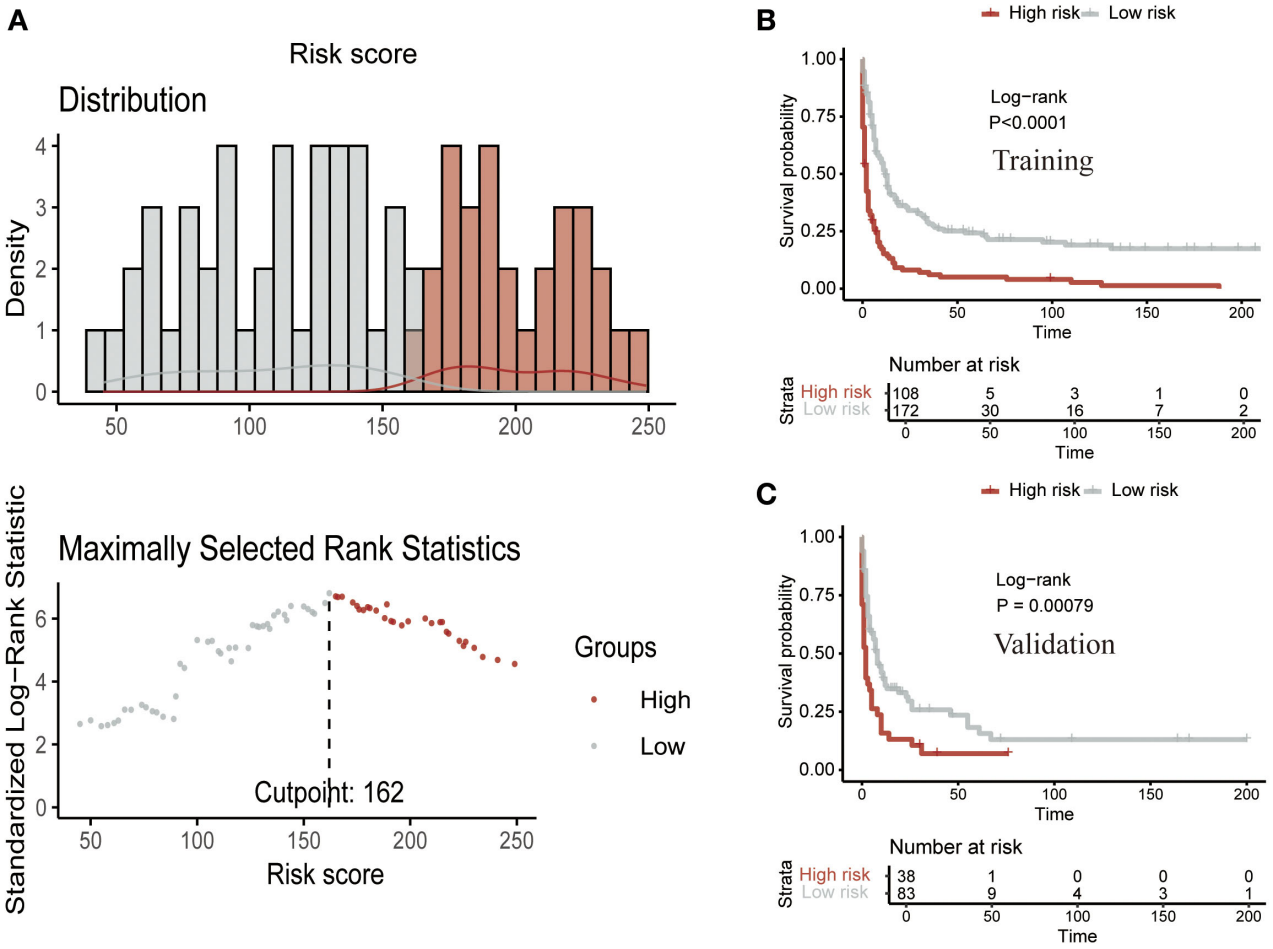
Risk stratification using the CS nomogram. **(A)** Nomogram-based risk score classification of patients into low- and high-risk groups based on survival probabilities. Kaplan-Meier survival analysis for the **(B)** training cohort and **(C)** validation cohort, demonstrating a significant survival difference between low- and high-risk groups.

Finally, we calculated the risk score for each patient based on the variable values from the nomogram. Using the optimal cutoff point of 162, patients were stratified into two risk groups: low-risk and high-risk (**Figure 8A**). Kaplan-Meier analysis further confirmed a significant prognostic difference (P<0.05) between these risk groups, both in the training (**Figure 8B**) and validation (**Figure 8C**) cohorts.

## 4 Discussion

EATL is a rare and aggressive form of T-cell non-Hodgkin lymphoma that primarily arises in the small intestine and is strongly associated with celiac disease (3, 5, 10). Due to its extremely low incidence, the study of EATL presents significant challenges, particularly in terms of obtaining large-scale data for comprehensive analysis. Given the poor prognosis of EATL, it is essential to conduct large-scale studies with long-term follow-up to better understand its incidence trends, prognostic factors, and survival outcomes. In this study, we attempted to fill these gaps by analyzing the incidence trends of EATL, identifying significant prognostic factors, and using CS analysis to develop a dynamic prognostic model. Through these analyses, we have gained valuable insights into the epidemiological characteristics and survival prognosis of EATL, offering a more accurate and clinically relevant tool for managing this challenging malignancy.

The incidence rate of EATL we calculated was 0.014 per 100,000. Two prior population-based studies from the United States reported incidence rates of 0.011 and 0.016 per 100,000 per year, while population-based studies from Europe reported rates of 0.04, 0.07, and 0.1 per 100,000 per year (10, 19–21). The analysis of EATL’s incidence trends revealed a concerning upward trajectory over the past two decades, with an APC of 2.63. Although EATL remains a rare disease, this increasing incidence could reflect greater awareness, improved diagnostic methods, or potentially an actual rise in cases. The extremely low incidence of EATL makes it difficult to track accurately, and this upward trend may indicate a broader recognition of the disease in clinical practice. The reasons for the increase in incidence remain speculative, but factors such as better reporting, advancements in diagnostic technologies (e.g., molecular techniques), and the growing recognition of its link to celiac disease may contribute to the observed trend. However, further investigation with large cohort studies is needed to establish the underlying causes definitively.

Our study identified several significant prognostic factors for OS in EATL patients, including age, tumor site, tumor site, tumor stage, surgery, and chemotherapy. Age and tumor stage were identified as independent risk factors, with older age and advanced tumor stage associated with poorer survival outcomes, consistent with previous studies (5, 8, 20, 22). The role of surgery and chemotherapy in prolonging survival highlighted the necessity of timely intervention and appropriate therapeutic strategies. And several studies have also highlighted the clinical efficacy of combining surgery with chemotherapy, which significantly improved the clinical outcomes of these tumors (8, 10, 23). However, despite treatment, the prognosis for EATL remains extremely poor, as reflected in the low 5-year survival rates. This reinforced the need for improved treatment protocols and the exploration of novel therapeutic strategies to address this aggressive lymphoma.

Given the dismal prognosis of EATL, we further explored its survival outcomes using CS analysis. This analysis allowed us to assess how survival probabilities change over time, providing a more nuanced understanding of the disease’s progression. Our findings revealed that the highest mortality rate occurs within the first year post-diagnosis, with survival probabilities significantly improving after surviving this critical period. This underscored the importance of early detection and intervention in EATL. The dynamic nature of CS analysis, which accounts for changing prognostic factors over time, offers valuable insights into the most critical time points for patient management. By identifying these high-risk periods, clinicians can better tailor treatment plans to improve survival chances during the most vulnerable phases of the disease.

The development of a nomogram model based on CS analysis represents a significant advancement in predicting survival outcomes for EATL patients (24). By integrating dynamic survival probabilities at different time points, this model provided a more accurate prediction of individual patient survival. The ability to stratify patients into low- and high-risk groups based on their predicted survival probabilities can guide clinical decision-making and help prioritize treatment interventions. This model’s clinical utility is particularly valuable, as it combines traditional prognostic factors with dynamic survival data, offering a comprehensive approach to managing EATL. The integration of CS analysis with the nomogram enhanced its accuracy and provided clinicians with a tool that reflected not only initial prognosis but also evolving survival probabilities over time (25–28).

Despite the strengths of our study, several limitations inherent to the SEER database should be acknowledged. First, in 2016 the WHO classification redefined monomorphic epitheliotropic intestinal T-cell lymphoma (MEITL) as a distinct entity, previously grouped with EATL. Because SEER lacks comprehensive histopathological details, particularly for cases diagnosed before 2016, a complete separation of these two entities was not feasible. Consequently, some MEITL cases may have been misclassified as EATL, potentially influencing our incidence and survival estimates. Second, the SEER database does not provide tumor-level biological information, including recurrent molecular alterations (SETD2, STAT5B, TP53), T-cell receptor subtype (γ/δ vs. α/β), or immunophenotypic markers such as CD8 and CD56. These features are increasingly recognized as clinically relevant for disease classification, treatment response, and prognosis in EATL/MEITL, but they could not be incorporated into our analysis or nomogram. Therefore, our findings are based mainly on demographic and clinicopathological characteristics. Future multi-institutional studies that integrate detailed pathological, immunophenotypic, and genomic data with outcomes are needed to refine risk stratification and develop biologically informed prognostic tools. Third, SEER lacks information on treatment details, including the timing and sequence of surgery (at diagnosis vs. later in the disease course, before vs. after chemotherapy) and chemotherapy regimen heterogeneity (specific agents, dosing schedules, or subsequent lines of therapy). This limited granularity prevents assessment of the prognostic impact of surgical timing, comparisons across chemotherapy regimens, or outcomes in patients who fail frontline therapy. Given the clinical relevance of these factors, future prospective or multi-institutional retrospective datasets with detailed therapeutic information will be critical for clarifying their influence on survival. In addition, SEER does not capture information on the presence of celiac disease or refractory celiac disease, which restricts our ability to explore the observed increase in EATL incidence. Finally, as a retrospective registry-based study, our findings are subject to potential biases and may not be generalizable to all populations. External validation of our model in independent cohorts and prospective studies will be essential to strengthen its clinical applicability.

Looking ahead, our findings lay the groundwork for the development of clinical decision-support tools to aid in the management of EATL. The CS–integrated nomogram we constructed demonstrated the feasibility of combining weighted clinicopathological variables with dynamic survival probabilities to generate individualized prognostic estimates. Such tools could be further refined by incorporating molecular alterations, immunophenotypic markers, treatment sequence, and regimen-specific details as these data become available in future multi-institutional datasets. Ultimately, integrating these enriched models into web-based platforms or electronic health record systems may provide clinicians with real-time, patient-specific prognostic information, thereby supporting risk-adapted treatment strategies, optimizing resource allocation, and improving patient counseling.

## Conclusions

Our study, utilizing the SEER database, provided valuable insights into the incidence trends, prognostic factors, and survival outcomes of EATL. The increasing incidence of EATL over the past two decades highlighted the growing recognition of this malignancy. We identified several independent prognostic factors, such as age, tumor site, tumor stage, surgery, and chemotherapy. Through CS analysis, we provided a more dynamic and nuanced understanding of the disease’s progression, demonstrating the critical importance of early intervention, particularly in the first year post-diagnosis. The development of a novel nomogram model, integrating dynamic survival data, offers clinicians a powerful tool for predicting individual survival outcomes and improving patient management.

## Data Availability

Publicly available datasets were analyzed in this study. This data can be found here: This data can be found in the SEER database (https://seer.cancer.gov/).
